# Supplementary figures and images for: Maternal Age, the Disparity across Regions and Their Correlation to Sudden Infant Death Syndrome in Taiwan: A Nationwide Cohort Study
Source: Children (Basel). 2021 Sep 1;8(9):771. doi: 10.3390/children8090771 (PMC8471108; doi:10.3390/children8090771)

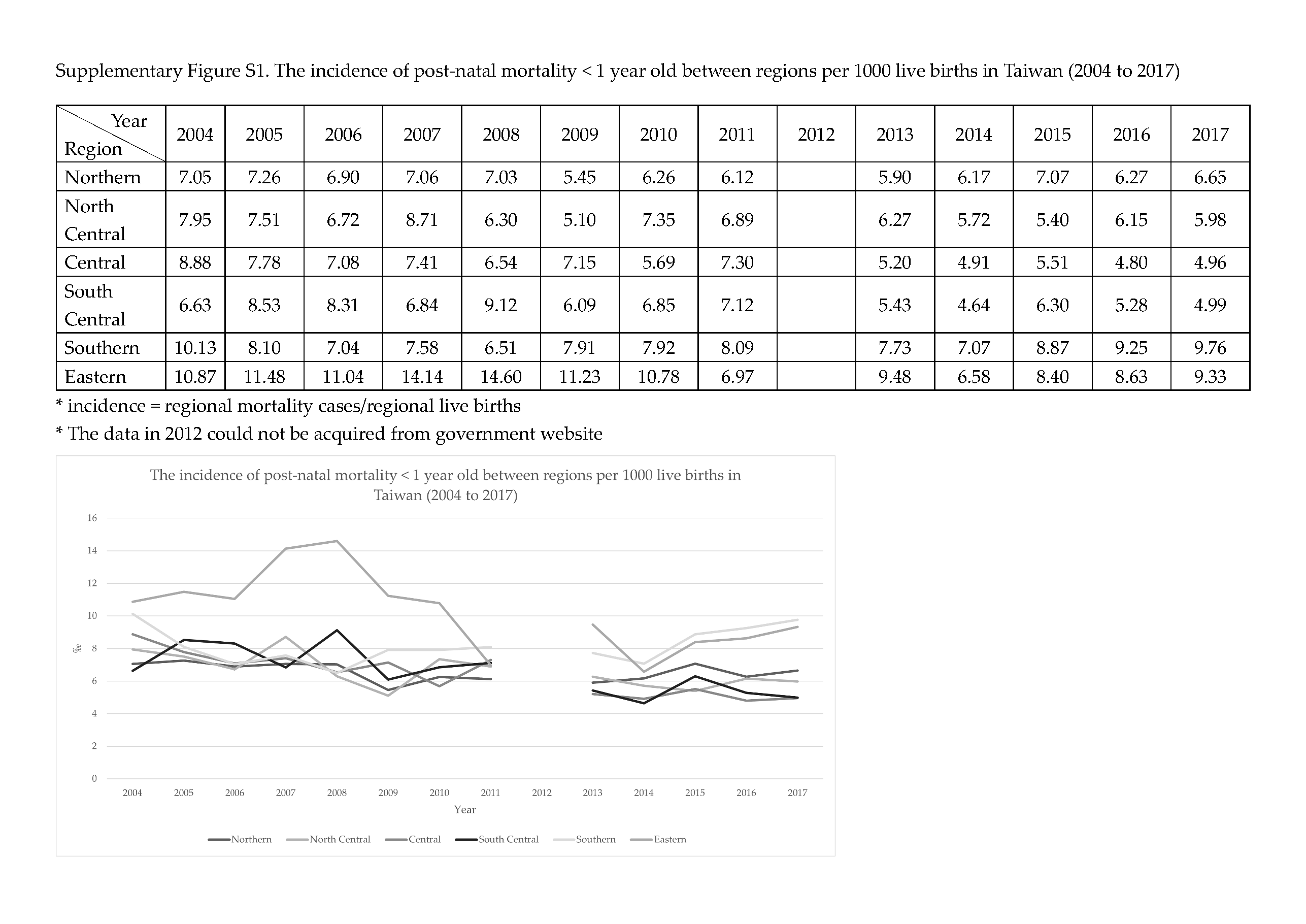

Supplement: Supplementary file 1 [file children-08-00771-s001.zip › children-1329847-supplementary.tiff]
